# Associations of hormone replacement therapy and oral contraceptives with risk of colorectal cancer defined by clinicopathological factors, beta-catenin alterations, expression of cyclin D1, p53, and microsatellite-instability

**DOI:** 10.1186/1471-2407-14-371

**Published:** 2014-05-25

**Authors:** Jenny Brändstedt, Sakarias Wangefjord, Björn Nodin, Jakob Eberhard, Karin Jirström, Jonas Manjer

**Affiliations:** 1 Department of Clinical Sciences, Lund, Oncology and Pathology, Lund University, Skåne University Hospital, Lund, Sweden; 2 Department of Surgery, Skåne University Hospital, Malmö, Sweden; 3 Department of Clinical Sciences, Malmö, Lund University, Skåne University Hospital, Malmö, Sweden; 4 Department of Plastic Surgery, Skåne University Hospital, Malmö, Sweden

## Abstract

**Background:**

Postmenopausal hormone therapy (HRT) and oral contraceptive (OC) use have in several studies been reported to be associated with a decreased colorectal cancer (CRC) risk. However, data on the association between HRT and OC and risk of different clinicopathological and molecular subsets of CRC are lacking. The aim of this molecular pathological epidemiology study was therefore to evaluate the associations between HRT and OC use and risk of specific CRC subgroups, overall and by tumour site.

**Method:**

In the population-based prospective cohort study Mamö Diet and Cancer, including 17035 women, 304 cases of CRC were diagnosed up until 31 December 2008. Immunohistochemical expression of beta-catenin, cyclin D1, p53 and MSI-screening status had previously been assessed in tissue microarrays with tumours from 280 cases. HRT was assessed as current use of combined HRT (CHRT) or unopposed oestrogen (ERT), and analysed among 12583 peri-and postmenopausal women. OC use was assessed as ever vs never use among all women in the cohort. A multivariate Cox regression model was applied to determine hazard ratios for risk of CRC, overall and according to molecular subgroups, in relation to HRT and OC use.

**Results:**

There was no significantly reduced risk of CRC by CHRT or ERT use, however a reduced risk of T-stage 1–2 tumours was seen among CHRT users (HR: 0.24; 95% CI: 0.09-0.77).

Analysis stratified by tumour location revealed a reduced overall risk of rectal, but not colon, cancer among CHRT and ERT users, including T stage 1–2, lymph node negative, distant metastasis-free, cyclin D1 - and p53 negative tumours.

In unadjusted analysis, OC use was significantly associated with a reduced overall risk of CRC (HR: 0.56; 95% CI: 0.44-0.71), but this significance was not retained in adjusted analysis (HR: 1.05: 95% CI: 0.80-1.37). A similar risk reduction was seen for the majority of clinicopathological and molecular subgroups.

**Conclusion:**

Our findings provide information on the relationship between use of HRT and OC and risk of clinicopathological and molecular subsets of CRC.

## Background

Colorectal cancer (CRC) is the third most common cancer in westernized countries with approximately 1.2 million new cases being diagnosed every year [1]. The incidence is higher among men than women, and this sex difference is likely related to hormonal factors. Indeed, observational and experimental evidence suggests that sex hormones, particularly oestrogen, play a role in colorectal cancer pathogenesis [2]. Yet, the effect of oestrogen on the risk of CRC is not fully understood. CRC comprises a heterogeneous group of diseases with different sets of genetic and epigenetic alterations that develop through different carcinogenetic pathways, characterized by distinctive models of genetic instability, subsequent clinical manifestations, and pathological characteristics. In order to understand how a particular exposure influences the carcinogenic process, it is of great importance that the exposure of interest is studied in relation to molecular alterations. Molecular pathologic epidemiology (MPE), first proposed in 2010 [2], is a multidisciplinary field that investigates the relationship between exposure factors with molecular signatures of the tumours.

In a large meta-analysis conducted in 1999, Grodstein *et al*. [3] found that hormone replacement therapy (HRT) use was associated with a decreased risk of colon cancer of approximately 35%. This association was further confirmed by the Women’s Health Initiative (WHI) Clinical Trial [4,5], a randomized, double-blind placebo controlled clinical trial, where intervention with oestrogen plus progestin yielded a 44% reduction in incident CRC, while oestrogen alone did not appear to affect CRC risk. The California Teachers study revealed that the risk for colon cancer was 36% lower among HRT users compared with never users, and the results did not differ by formulation [6]. Further, the risk was lower among recent HRT users with increasing duration between 5 and 15 years of use, but this risk reduction was was not seen in the longest duration group (more than 15 years of use) [6]. A meta-analysis of 18 observational studies showed a 20% reduction in colon cancer incidence among women having ever used HRT, and duration of HRT use did not influence risk estimates [7].

Hence, while epidemiological data support a protective effect of HRT on CRC, the associations between different combinations of HRT and CRC risk remain unclear. The results from the WHI, wherein unopposed estrogen did not appear to affect CRC risk, imply an important protective role of progestins, but the biological mechanisms underlying the effect of progestins in the colorectum are not well understood.

Colorectal carcinogenesis can be regarded as a complex process involving multiple genetic and epigenetic alterations [8,9]. Accumulating evidence suggests that the influence of aetiological factors may differ according to the carcinogenetic pathway. As traditional cancer epidemiology-approaches have not generally taken clinicopathological and key molecular characteristics, e.g. expression of beta-catenin, cyclin D1, p53 and mismatch repair proteins [10-13] into account, the impact of hormonal factors on CRC risk may be further clarified by doing so [14]. So far, studies on associations of HRT and molecular subgroups of CRC have been limited and inconsistent [15-17].

The epidemiological evidence for an association between oral contraceptives (OC) and CRC risk is also somewhat inconsistent in that some studies have suggested inverse associations [18-22], whereas others have found no associations [23-26]. A recent meta-analysis, summarising the results from seven cohort- and eleven case–control studies, reported a statistically significant risk reduction of 19% among ever users of OC compared with never users, although there was no clear risk reduction with increasing duration of use [27].

Taken together, the findings from these observational and experimental studies suggest that exogenous sex hormones may play an important role in colorectal carcinogenesis. The aim of this study was therefore to investigate the associations of postmenopausal HRT (combinations with oestrogen and progesterone as well as use of oestrogen alone) and OC use with CRC risk in the Malmö Diet and Cancer Study (MDCS), a large proscpective population based cohort study. In particular, we examined risk of CRC according to tumour site, TNM-stage, expression of beta-catenin-, p 53 and cyclin D1, and microsatellite instability (MSI) screening status.

## Methods

### The malmö diet and cancer study

The Malmö Diet and Cancer Study (MDCS) is a population-based prospective cohort study of male and female residents in Malmö, Sweden, enrolled between 1991 and 1996. At the end of baseline examinations the total female cohort consisted of 17035 women, born 1923– 1950 [28]. A questionnaire assessed education, reproductive factors, exposure to OC, HRT, alcohol consumption and smoking habits. Weight and length was measured by a trained nurse and body mass index (BMI) was calculated as kg/m2. Information on gynaecological surgery was retrieved from hospital records. Ethical permission for the MDCS (Ref. 51/90), and the present study (Ref. 530/2008), was obtained from the Ethics Committee at Lund University. Written informed consent was obtained from each participant.

### Exposure assessement

Use of HRT was assessed in two ways. All participants were asked to keep a diary of medications. Moreover, medications were recorded in a questionnaire using an open-ended question on current use. Medications were coded according to the ATC classification. The present study has divided use of HRT into oestrogen alone (ERT), and combined HRT (CHRT), assessed as current use or not. The use of oral contraceptives was assessed as ever versus never use.

### Study population

In total, 17035 women were included in the female cohort [28]. A woman was considered postmenopausal if she had undergone (I) bilateral oophorectomy or (II) hysterectomy, without bilateral oophorectomy, and if she was 55years of age or (III) if the above criteria were absent and she affirmed that her menstruations had ceased at least during 2years prior to baseline examinations.

A total of 12 583 (73.9%) women classified as peri- or postmenopausal at baseline made up for the study population in all HRT analyses. However, in the analyses related to OC, both pre-, peri- and postmenopausal women were included in the analysis, i.e. the entire female cohort.

### End-point retrieval

Incident cases of invasive CRC in the MDCS were identified through the Swedish Cancer Registry and vital status was determined by record linkage with the Swedish Cause of Death Registry. End of follow-up was 31 December 2009. Information on vital status and cause of death was obtained from the Swedish Cause of Death Registry until 31 December 2009. Time on study was defined as time from baseline to diagnosis, death or end of follow-up 31 December 2009. Median time from baseline until diagnosis was 8.6 (SD = 4.3) years and the median follow-up time in the entire cohort was 13.7 (SD = 3.2) years.

### Tumour characteristics

Patient and tumour characteristics in the entire cohort have been described in detail previously [29-31]. In the female cohort, a total of 304 incident invasive CRC cases were identified. Forty-five cases were diagnosed with CRC before baseline examination, i.e. prevalent colorectal cancers, and therefore excluded from the study. Cases with other prevalent cancers were not excluded from the study. Of all incident CRC cases, 180 (59.2%) had tumours located in the colon and 107 (35.2%) had tumours located in the rectum. All tumours were histopathologically re-evaluated by a senior pathologist (KJ).

According to the TNM classification, 33 (10.8%) cases presented in T-stage 1, 30 (9.8%) in T-stage 2, 159 (52.3%) in T-stage 3 and 47 (15.4%) in T-stage 4. One hundred and fifty two (50%) cases presented with lymph node negative (N0) disease, 73 (24.0%) had N1 (1–3 positive lymph nodes) and 33 (10.9%) N2 (4 or more positive lymph nodes) disease. Two hundred and thirty seven (78%) patients did not have distant metastases (M0), and 56 (18.4%) had M1 disease.

### Tissue microarray construction and immunohistochemistry

Tissue microarrays (TMAs) had been constructed as previously described [29,30]. In brief, two 1.0 mm cores were taken from each tumour and mounted in a new recipient block using a semi-automated arraying device (TMArrayer, Pathology Devices, Westminster, MD, USA). Among the 304 incident CRC cases in the cohort, a total number of 280 tumours were suitable for TMA-construction, and as demonstrated previously, there was no selection bias regarding the distribution of clinicopathological characteristics between the TMA cohort and the full cohort [29].

For immunohistochemical analysis, 4 μm TMA-sections were automatically pre-treated using the PT-link system (DAKO, Glostrup, Denmark) and then stained in an Autostainer Plus (DAKO, Glostrup, Denmark). MSI screening status was evaluated as previously described [32], whereby tumour samples lacking nuclear staining of mismatch repair proteins MLH1, PMS2, MSH2 or MSH6 were considered to have a positive MSI screening status. Hereafter, tumours with a positive MSI screening status are referred to as MSI and tumours with negative MSI screening status are referred to as MSS.

Immunohistochemical staining of beta-catenin was performed and evaluated as previously described [33], whereby membranous staining was denoted as 0 (present) or 1 (absent), cytoplasmic staining intensity as 0–2 and nuclear staining intensity as 0–2. In this study, the analyses were limited to nuclear expression of beta-catenin. Cyclin D1 expression was evaluated as previously described [30] and p53 positivity was defined as > =50% tumour cells with strong nuclear staining intensity in accordance with previous studies [34].

### Statistical methods

A Cox proportional hazards analysis was applied in order to compare risk of CRC and CRC subgroups between ERT-, CHRT- and non HRT users, as well as between OC users and non OC users. For the subtype-specific analyses, the outcome variable was incident CRC with the molecular marker of interest; all other incident CRCs (including those with missing or unknown values for the molecular marker of interest) were considered censored observations at the date of diagnosis. This yielded relative risks (HR) with a 95% confidence interval. Time on study was used as the underlying time scale, defined as time from baseline to diagnosis, death or end of follow-up 31 December 2009. The proportional hazards assumption was confirmed by a log, − log plot [35]. Chi square test was applied for assessment of the distribution of investigative factors according to baseline characteristics. A case-to-case analysis examined the heterogeneity between different tumour subgroups regarding their association to anthropometric factors using an unconditional logistic regression model.

In the multivariate Cox analysis potential confounders were included, i.e. age (years), educational level, smoking habits, alcohol consumption and BMI (Table 1). All statistical analyses were conducted using SPSS version 20 (SPSS Inc., Chicago, IL, USA). A two-tailed p-value less than 0.05 was regarded as statistically significant.

**Table 1:** Distribution of risk factors in relation to HRT, ERT, and OC use

**Factor (n of subjects with information)**	**Category**	**Current CHRT n = 3051 (18.0%)**	**Current ERT n = 1375 (8.0%)**	**HRT non use n = 12519 (73.9%)**	**Ever OC n = 8353 (49.1%)**	**Never OC n = 8661 (50.1%)**
Age at baseline (16990)	Years*	*56.7*	*59.2*	*57.6*	*53.7*	*61.0*
p				*<0.001*		*<0.001*
Education (16947)	Not completed school	0.2’	0.2	0.9	0.3	1.2
	Elementary school (6–8 years)	31.8	34.7	40.2	29.6	47.4
	0-level college (9–10 years)	33.4	34.4	29.7	32.5	28.3
	A-level college (10–12 years)	7.8	6.2	6.8	8.1	5.8
	1 year university	8.9	8.6	8.2	9.9	6.9
	University degree	17.6	15.9	14.2	19.5	10.3
p				<0.001		<0.001
Smoking status (16984)	Yes regularly	23.7	20.3	23.7	27.8	20.6
	Yes occasionally	4.8	4.0	4.2	4.9	3.8
	Former smoker	31.2	18.0	26.9	31.6	25.0
	Never smoked	40.0	44.7	45.2	35.8	54.2
p				<0.001		<0.001
Alcohol consumption (16990)	g/day*	*9.47*	*8.65*	*7.27*	*9.3*	*6.05*
p				*<0.001*		*<0.001*
BMI (16964)	*Kg/m*^2*^	*24.8*	*25.0*	*26.6*	*24.8*	*26.0*
*p*				*<0.001*		*<0.001*

*All values given in percentages unless otherwise indicated.

## Results

### Baseline characteristics

The distribution of risk factors according to use and non use of HRT and OC is shown in Table 1. There were significant differences in the distribution of age, educational level, smoking status, alcohol consumption and BMI among users and non users of HRT and OC. Non HRT users were more often never smokers (p = <0.001), had a lower level of education (p = <0.001), consumed less alcohol (p = <0.001), had a higher BMI (p = <0.001), and a higher age (p = <0.001), than HRT users. Additionally, ever users of OC were younger (p = <0.001), had a higher level of education (p = <0.001), were more often smokers (p = <0.001), consumed more alcohol (p = <0.001), and had a lower BMI (p = <0.001), compared with never users.

### HRT use and risk of colorectal cancer subgroups

There were no statistically significant associations between HRT use, combined (CHRT) or estrogen only (ERT), and overall CRC risk (Table 2). We found a significantly reduced risk of T stage 1 and 2 tumours among current users of CHRT (HR: 0.30; 95% CI: 0.09-0.96). (Table 2). There were no associations between neither CHRT nor ERT use and risk of other particular subgroups of CRC (Tables 2 and 3).

**Table 2:** Risk of colorectal cancer, overall and according to clinicopathological factors, in relation to HRT and ERT use

**Tumour subgroups**	**HRT use**	**Number of cases**	**HR crude**	***p***	**HR adjusted**	***p***
CRC	No	236	1.00		1.00	
	ERT	24	0.89(0.59-1.35)	*0.583*	0.94(0.62-1.44)	*0.788*
	CHRT	41	0.74(0.53-1.03)	*0.076*	0.94(0.67-1.31)	*0.701*
T stage 1 & 2	No	54	1.00		1.00	
	ERT	3	0.53(0.17-1.70)	*0.284*	0.56(0.17-1.80)	*0.560*
	CHRT	3	0.24(0.09-0.77)	*0.016*	0.30(0.09-0.96)	*0.043*
T stage 3 & 4	No	156	1.00		1.00	
	ERT	17	0.93(0.56-1.52)	*0.761*	1.01(0.61-1.67)	*0.966*
	CHRT	32	0.87(0.59-1.27)	*0.471*	1.14(0.77-1.68)	*0.511*
N0	No	119	1.00		1.00	
	ERT	14	1.03(0.59-1.79)	*0.912*	1.15(0.66-2.01)	*0.615*
	CHRT	22	0.79(0.50-1.24)	*0.302*	1.05(0.66-1.67)	*0.838*
N1 & N2	No	79	1.00		1.00	
	ERT	7	0.75(0.35-1.63)	*0.471*	0.78(0.36-1.68)	*0.522*
	CHRT	15	0.81(0.47-1.40)	*0.449*	0.95(0.54-1.67)	*0.860*
M0	No	181	1.00		1.00	
	ERT	20	0.96(0.61-1.52)	*0.863*	1.04(0.66-1.66)	*0.861*
	CHRT	34	0.80(0.55-1.15)	*0.232*	1.03(0.71-1.49)	*0.893*
M1	No	45	1.00		1.00	
	ERT	4	0.78(028–2.16)	*0.632*	0.77(0.28-2.16)	*0.625*
	CHRT	7	0.66(0.30-1.47)	*0.311*	0.80(0.36-1.80)	*0.589*

Adjusted for age, bmi, educational level, smoking habits and alcohol consumption.

**Table 3:** Risk of colorectal cancer and molecular subgroups in relation to HRT and ERT use

**Tumour subgroups**	**HRT use**	**n**	**HR crude**	***p***	**HR adjusted**	**p**
beta-catenin +	No	126	1.00		1.00	
	ERT	13	0.93(0.52-1.64)	*0.790*	1.04(0.58-1.84)	*0.902*
	CHRT	19	0.64(0.40-1.04)	*0.074*	0.87(0.53-1.42)	*0.868*
beta-catenin −	No	80	1.00		1.00	
	ERT	7	0.76(0.35-1.65)	*0.490*	0.77(0.36-1.68)	*0.516*
	CHRT	13	0.69(0.39-1.24)	*0.217*	0.83(0.46-1.50)	*0.533*
cyclin D1 +	No	173	1.00		1.00	
	ERT	16	0.81(0.49-1.35)	*0.416*	0.87(0.52-145)	*0.591*
	CHRT	29	0.72(0.48-1.06)	*0.096*	0.93(0.63-1.39)	*0.734*
cyclin D1 −	No	31	1.00		1.00	
	ERT	4	1.16(0.41-3.28)	*0.782*	1.26(0.44-3.59)	*0.661*
	CHRT	5	0.68(0.27-1.75)	*0.425*	0.84(0.32-2.19)	*0.721*
p53 +	No	99	1.00		1.00	
	ERT	11	0.98(0.53-1.83)	*0.956*	1.07(0.57-1.99)	*0.843*
	CHRT	18	0.78(0.47-1.29)	*0.334*	1.00(0.59-1.65)	*0.970*
p53 −	No	107	1.00		1.00	
	ERT	9	0.74(0.37-1.45)	*0.374*	0.79(0.40-1.55)	*0.488*
	CHRT	16	0.63(0.38-1.07)	*0.089*	0.84(0.49-1.42)	*0.508*
MSI	No	38	1.00		1.00	
	ERT	1	0.23(0.03-1.63)	*0.140*	0.24(0.03-1.72)	*0.154*
	CHRT	5	0.56(0.22-1.43)	*0.225*	0.80(0.31-2.06)	*0.649*
MSS	No	164	1.00		1.00	
	ERT	18	0.97(0.60-1.58)	*0.903*	1.06(0.65-1.72)	*0.829*
	CHRT	28	0.73(0.49-1.09)	*0.120*	0.92(0.61-1.39)	*0.700*

Adjusted for age, bmi, educational level, smoking habits and alcohol consumption.

When stratifying for cancer site, our results indicated significant associations of HRT use and an overall reduced risk of rectal, but not colon, cancer (HR: 0.32; 95% CI: 0.14-0.71). Moreover, HRT use was significantly associated with T stage 1 and 2 (HR: 0.03; 95%: 0.00-0.36), lymph node negative (HR: 0.22; 95% CI: 0.06-0.77) and non-metastatic (HR: 0.42; 95% CI: 0.18-0.98) rectal, but not colon, cancer (Table 4). We also found significant associations between HRT and cyclin D1 negative- (HR: 0.07; 95% CI: 0.01-0.88) and p53 negative tumours (HR: 0.19; 95% CI: 0.04-0.96) in the rectum (Table 5). Heterogeneity analysis revealed no significance between tumour subgoups.

**Table 4:** Risk of CRC and clinicopathological subgrops in relation to HRT and ERT use in colon and rectum

		**Colon**					**Rectum**				
**Tumour subgroups**	**HRT use**	**n**	**HR crude**	***p***	**HR adjusted**	***p***	**n**	**HR crude**	***p***	**HR adjusted**	***p***
CRC	No	150	1.00		1.00		81	1.00		1.00	
	ERT	15	1.21(0.71-2.06)	*0.488*	1.17(0.67-2.02)	*0.584*	8	0.49(0.23-1.03)	*0.061*	0.38(0.17-0.86)	*0.020*
	CHRT	28	1.04(0.68-1.57)	*0.873*	1.16(0.74-1.80)	*0.521*	13	0.44(0.23-0.86)	*0.017*	0.32(0.14-0.71)	*0.005*
T stage 1 & 2	No	27	1.00		1.00		24	1.00		1.00	
	ERT	1	0.52(0.07-3.89)	*0.525*	0.34(0.04-2.70)	*0.308*	2	0.32(0.07-1.45)	*0.138*	0.11(0.02-0.67)	*0.017*
	CHRT	1	0.21(0.03-1.54)	*0.125*	0.24(0.03-1.90)	*0.178*	2	0.09(0.01-0.76)	*0.026*	0.03(0.00-0.36)	*0.031*
T stage 3 & 4	No	117	1.00		1.00		42	1.00		1.00	
	ERT	13	1.29(0.73-2.30)	*0.381*	1.30(0.72-2.35)	*0.384*	4	0.53(0.19-1.48)	*0.223*	0.51(0.17-1.55)	*0.233*
	CHRT	24	1.17(0.75-1.83)	*0.486*	1.27(0.78-2.04)	*0.335*	8	0.72(0.33-1.58)	*0.414*	0.74(0.28-1.94)	*0.533*
N0	No	72	1.00		1.00		45	1.00		1.00	
	ERT	11	1.72(0.91-3.26)	*0.096*	1.54(0.79-3.00)	*0.203*	3	0.36(0.11-1.20)	*0.097*	0.31(0.08-1.13)	*0.076*
	CHRT	16	1.20(0.70-2.08)	*0.508*	1.49(0.83-2.67)	*0.183*	6	0.29(0.10-0.85)	*0.024*	0.22(0.06-0.77)	*0.222*
N1 & N2	No	64	1.00		1.00		18	1.00		1.00	
	ERT	4	0.76(0.28-2.10)	*0.596*	0.79(0.28-2.23)	*0.659*	3	1.03(0.30-3.56)	*0.959*	1.00(0.26-3.92)	*1.000*
	CHRT	10	0.90(0.46-1.77)	*0.762*	0.79(0.37-1.65)	*0.522*	5	1.18(0.42-3.34)	*0.757*	1.00(0.27-3.73)	*1.000*
M0	No	114	1.00		1.00		64	1.00		1.00	
	ERT	13	1.38(0.77-2.46)	*0.277*	1.25(0.69-2.26)	*0.471*	7	0.54(0.24-1.20)	*0.130*	0.43(0.18-1.03)	*0.057*
	CHRT	22	1.10(0.69-1.74)	*0.695*	1.21(0.74-1.97)	*0.446*	12	0.54(0.29-1.09)	*0.084*	0.42(0.18-0.98)	*0.044*
M1	No	32	1.00		1.00		13	1.00		1.00	
	ERT	3	1.04(0.32-3.40)	*0.953*	1.24(0.37-4.16)	*0.732*	1	0.31(0.04-2.48)	*0.269*	0.32(0.03-3.24)	*0.335*
	CHRT	6	1.06(0.44-2.56)	*0.900*	1.13(0.42-3.00)	*0.809*	1	0.14(0.02-1.22)	*0.075*	0.06(0.00-1.18)	*0.064*

Adjusted for age, bmi, educational level, smoking habits and alcohol consumption.

**Table 5:** Risk of CRC and molecular subgroups in relation to HRT and ERT use in colon and rectum

		**Colon**					**Rectum**				
**Tumour subgroups**	**HRT use**	**n**	**HR crude**	***p***	**HR adjusted**	***p***	**n**	**HR crude**	***p***	**HR adjusted**	***p***
beta-catenin +	No	68	1.00		1.00		51	1.00		1.00	
	ERT	9	1.62(0.80-3.26)	*0.180*	1.52(0.74-3.16)	*0.257*	4	0.38(0.14-1.09)	*0.073*	1.00(0.45-2.25)	*1.000*
	CHRT	11	0.88(0.46-1.67)	*0.696*	0.96(0.48-1.91)	*0.910*	8	0.44(0.19-1.02)	*0.054*	1.00(0.44-2.26)	*1.000*
beta-catenin −	No	64	1.00		1.00		17	1.00		1.00	
	ERT	4	0.76(0.27-2.09)	*0.588*	0.71(0.25-1.97)	*0.506*	3	0.87(0.24-3.12)	*0.833*	1.00(0.27-3.73)	*1.000*
	CHRT	10	0.91(0.46-1.78)	*0.779*	0.92(0.45-1.88)	*0.808*	3	0.41(0.09-1.83)	*0.242*	1.00(0.25-3.95)	*1.000*
cyclin D1 +	No	120	1.00		1.00		50	1.00		1.00	
	ERT	10	1.04(0.54-1.98)	*0.917*	0.96(0.49-1.86)	*0.895*	6	0.56(0.23-1.34)	*0.189*	0.44(0.17-1.14)	*0.091*
	CHRT	18	0.86(0.52-1.42)	*0.557*	0.95(0.56-1.62)	*0.854*	11	0.60(0.28-1.28)	*0.188*	0.51(0.21-1.24)	*0.135*
cyclin D1 −	No	15	1.00		1.00		16	1.00		1.00	
	ERT	3	2.05(0.59-7.10)	*0.260*	2.34(0.65-8.44)	*0.196*	1	0.37(0.05-2.81)	*0.334*	0.28(0.03-2.27)	*0.232*
	CHRT	4	1.33(0.44-4.05)	*0.615*	1.50(0.46-4.94)	*0.504*	1	0.19(0.02-1.56)	*0.124*	0.07(0.01-0.88)	*0.039*
p53 +	No	54	1.00		1.00		40	1.00		1.00	
	ERT	6	1.38(0.59-3.24)	*0.454*	1.27(0.53-3.02)	*0.588*	5	0.54(0.21-1.41)	*0.208*	1.00(0.43-2.35)	*1.000*
	CHRT	9	0.94(0.46-1.92)	*0.869*	1.02(0.48-2.20)	*0.951*	9	0.56(0.25-1.27)	*0.168*	1.00(0.42-2.40)	*1.000*
p53 −	No	82	1.00		1.00		26	1.00		1.00	
	ERT	7	1.02(0.47-2.21)	*0.968*	0.95(0.43-2.11)	*0.903*	2	0.47(0.11-2.02)	*0.308*	0.23(0.05-1.18)	*0.078*
	CHRT	13	0.89(0.49-1.61)	*0.703*	1.00(0.54-1.86)	*0.996*	3	0.36(0.09-1.54)	*0.170*	0.19(0.04-0.96)	*0.044*
MSI	No	36	1.00		1.00		2	1.00		1.00	
	ERT	1	0.36(0.05-2.67)	*0.320*	0.28(0.04-2.08)	*0.212*	0	-		-	
	CHRT	5	0.84(0.32-2.16)	*0.710*	0.90(0.34-2.40)	*0.832*	0	-		-	
MSS	No	97	1.00		1.00		64	1.00		1.00	
	ERT	11	1.35(0.72-2.53)	*0.349*	1.36(0.71-2.60)	*0.352*	7	0.54(0.24-1.22)	*0.138*	1.00(0.49-2.03)	*1.000*
	CHRT	17	0.98(0.58-1.65)	*0.943*	1.08(0.62-1.89)	*0.789*	11	0.46(0.22-0.97)	*0.041*	1.00(0.49-2.05)	*1.000*

Adjusted for age, bmi, educational level, smoking habits and alcohol consumption.

### Oral contreceptive use and risk of colorectal cancer subgroups

As demonstrated in Table 6, there was a statistically significant inverse association between ever-use of OC and overall CRC risk (HR: 0.56; 95% CI: 0.44-0.71) in unadjusted analysis. However, after adjustment for well-established risk factors of CRC, i.e. age, BMI, alcohol consumption, smoking habits and educational level, this significant association was lost.

**Table 6:** Risk of colorectal cancer, overall and according to clinicopathological factors, in relation to oral contraceptive (OC) use

**Tumour subgroups**	**OC use**	**n**	**HR crude**	***p***	**HR adjusted**	***p***
CRC	No	194	1.00		1.00	
	OC	107	0.56(0.44-0.71)	*<0.001*	1.05(0.80-1.37)	*0.738*
T stage 1 & 2	No	45	1.00		1.00	
	OC	17	0.39(0.22-0.68)	*0.001*	0.69(0.37-1.28)	*0.239*
T stage 3 & 4	No	127	1.00		1.00	
	OC	77	0.62(0.47-0.82)	*0.001*	1.21(0.87-1.67)	*0.254*
N0	No	105	1.00		1.00	
	OC	46	0.45(0.32-0.63)	*<0.001*	0.82(0.56-1.22)	*0.328*
N1 & N2	No	60	1.00		1.00	
	OC	45	0.78(0.52-1.13)	*0.176*	1.36(0.88-2.11)	*0.173*
M0	No	150	1.00		1.00	
	OC	85	0.58(0.44-0.75)	*<0.001*	1.05(0.78-1.42)	*0.744*
M1	No	38	1.00		1.00	
	OC	17	0.46(0.26-0.81)	*0.007*	0.90(0.47-1.70)	*0.734*
beta-catenin+	No	105	1.00		1.00	
	OC	51	0.50(0.35-0.70)	*<0.001*	0.97(0.66-1.41)	*0.853*
beta-catenin−	No	61	1.00		1.00	
	OC	42	0.70(0.47-1.04)	*0.076*	1.31(0.84-2.05)	*0.232*
cyclin D1+	No	138	1.00		1.00	
	OC	75	0.55(0.42-0.74)	*<0.001*	1.16(0.85-1.60)	*0.351*
cyclin D1−	No	30	1.00		1.00	
	OC	15	0.51(0.27-0.94)	*0.032*	0.63(0.32-1.27)	*0.199*
p53+	No	81	1.00		1.00	
	OC	46	0.58(0.41-0.83)	*0.003*	1.06(0.70-1.60)	*0.744*
p53−	No	88	1.00		1.00	
	OC	45	0.52(0.36-0.74)	*<0.001*	1.04(0.70-1.56)	*0.840*
MSI	No	28	1.00		1.00	
	OC	17	0.62(0.34-1.13)	*0.117*	1.78(0.92-3.47)	*0.088*
MSS	No	136	1.00		1.00	
	OC	74	0.56(0.42-0.74)	*<0.001*	0.98(0.71-1.36)	*0.912*

Adjusted for age, bmi, educational level, smoking habits and alcohol consumption.

Similar statistically significant inverse associations were seen between OC use and the majority of clinicopathological and molecular subgroups, except for lymph node positive disease, negative nuclear betacatenin expression and MSS tumours (Table 6). Again, no statistically significant results were seen after adjustment for established risk factors.

In the analysis stratified for cancer site, a significantly increased risk was found of lymph node positive (HR: 1.81 95% CI: 1.00-3.28) and non-metastatic disease (HR: 1.55; 95% CI: 1.00-2.40), as well as for cyclin D1 positive tumours (HR: 1.62 95% CI: 1.04-2.51) in the colon (Table 7). No associations were found between OC use and specific subgroups of rectal cancer, or overall risk for colon or rectal cancer. No significant heterogeneity was found between tumour subgoups.

**Table 7:** Risk of colorectal cancer, overall and according to clinicopathological factors, in relation to oral contraceptive (OC) use in colon and rectum

		**Colon**					**Rectum**				
**Tumour subgroups**	**OC use**	**n**	**HR crude**	***p***	**HR adjusted**	***p***	**n**	**HR crude**	***p***	**HR adjusted**	***p***
CRC	No	116	1.00		1.00		69	1.00		1.00	
	OC	59	1.00(0.73-1.37)	*0.998*	1.41(0.96-2.08)	*0.080*	37	1.02(0.68-1.53)	*0.934*	0.97(0.59-1.60)	*0.900*
T stage 1&2	No	22	1.00		1.00		21	1.00		1.00	
	OC	8	0.72(0.32-1.62)	*0.422*	1.43(0.56-3.66)	*0.462*	8	0.76(0.33-1.76)	*0.521*	0.67(0.23-1.89)	*0.445*
T stage 3 &4	No	89	1.00		1.00		34	1.00		1.00	
	OC	49	1.08(0.76-1.54)	*0.655*	1.43(0.93-2.22)	*0.107*	24	1.22(0.72-2.07)	*0.460*	1.27(0.64-2.53)	*0.488*
N0	No	63	1.00		1.00		37	1.00		1.00	
	OC	26	0.77(0.49-1.22)	*0.271*	1.17(0.67-2.03)	*0.582*	18	0.93(0.52-1.66)	*0.811*	0.90(0.43-1.88)	*0.780*
N1 & N2	No	43	1.00		1.00		17	1.00		1.00	
	OC	30	1.38(0.86-2.20)	*0.181*	1.81(1.00-3.28)	*0.051*	12	1.20(0.57-2.52)	*0.630*	1.00(0.39-2.56)	*1.000*
M0	No	88	1.00		1.00		57	1.00		1.00	
	OC	47	1.05(0.73-1.49)	*0.804*	1.55(1.00-2.40)	*0.050*	31	1.00(0.64-1.56)	*0.999*	0.83(0.47-1.45)	*0.507*
M1	No	26	1.00		1.00		9	1.00		1.00	
	OC	12	0.90(0.45-1.80)	*0.771*	0.96(0.41-2.30)	*0.955*	5	1.09(0.35-3.36)	*0.880*	1.75(0.44-6.86)	*0.425*
beta-catenin+	No	56	1.00		1.00		43	1.00		1.00	
	OC	24	0.84(0.52-1.37)	*0.489*	1.36(0.75-2.47)	*0.308*	24	1.02(0.62-1.70)	*0.931*	1.00(0.53-1.88)	*1.000*
beta-catenin−	No	45	1.00		1.00		14	1.00		1.00	
	OC	29	1.27(0.79-2.03)	*0.322*	1.70(0.97-3.00)	*0.065*	10	1.41(0.61-3.24)	*0.421*	1.00(0.35-2.87)	*1.000*
cyclin D1+	No	88	1.00		1.00		44	1.00		1.00	
	OC	46	1.04(0.72-1.47)	*0.843*	1.62(1.04-2.51)	*0.032*	24	1.06(0.64-1.76)	*0.826*	1.04(0.56-1.95)	*0.893*
cyclin D1−	No	15	1.00		1.00		14	1.00		1.00	
	OC	8	1.00(0.42-2.36)	*0.999*	0.90(0.31-2.60)	*0.844*	7	0.82(0.33-2.03)	*0.666*	0.77(0.24-2.48)	*0.660*
p53+	No	45	1.00		1.00		32	1.00		1.00	
	OC	20	0.90(0.52-1.53)	*0.685*	1.28(0.67-2.45)	*0.451*	22	1.37(0.78-2.39)	*0.274*	1.00(0.49-2.02)	*1.000*
p53−	No	59	1.00		1.00		26	1.00		1.00	
	OC	34	1.11(0.73-1.70)	*0.625*	1.54(0.90-2.63)	*0.115*	9	0.59(0.28-1.27)	*0.177*	0.52(0.21-1.30)	*0.161*
MSI	No	25	1.00		1.00		2	1.00		1.00	
	OC	14	1.08(0.56-2.09)	*0.809*	1.85(0.82-1.18)	*0.139*	0	-		-	
MSS	No	76	1.00		1.00		54	1.00		1.00	*1.000*
	OC	39	1.02(0.69-1.50)	*0.938*	1.43(0.89-2.32)	*0.143*	32	1.10(0.70-1.72)	*0.678*	1.00(0.58-1.74)	

Adjusted for age, bmi, educational level, smoking habits and alcohol consumption.

## Discussion

In this prospective cohort study, we present data on associations between use of HRT and OC and overall risk of CRC, as well as risk of different clinicopathological and molecular subgroups thereof. It should be pointed out that, due to the limited number of cases available in the subgroup analyses, the associations were rather modest. In the present study, we could not demonstrate the expected risk reduction of HRT use and CRC use, which is in contrast with findings from several previous studies [3,36-39], as well as with three large meta-analyses, demonstrating a significantly lower incidence of CRC with use of combined hormone therapy [7,40,41]. As regards associations with TNM stage in the entire cohort, we found a significantly reduced risk of T-stage 1 and 2 tumours, but not of any other particular subgroups of CRC. When stratifying for cancer site, we found a significant risk reduction for CHRT use and overall risk of rectal, but not colon, cancer. Significant associations were also seen between CHRT use and risk of T-stage 1 and 2 tumours, lymph node negativity, non metastatic disease, cyclin D1 negativity and p53 negativity for tumours located in the rectum. Again, it must be emphasized that these results are based on a very limited number of cases. Previous studies on HRT and risk of colon and rectal cancer, respectively, are sparse and present diverging results. Most studies report a similar risk reduction for both colon- and rectal cancer [40-42], however at least two studies reported a significantly lower risk of colon, but not rectal, cancer, with the use of HRT [6,37]. The potential biological mechanisms underlying the differences in risk related to location remain unclear.

The most frequently cited study on this subject is the Women’s Health Initiative Trial [4], that demonstrated a 44% risk reduction with continous combined estrogen plus progesterin therapy compared to the placebo group, whereas unopposed estrogen therapy was not associated with a decreased CRC risk. However, despite the convincing risk reduction of 44%, the WHI clinical trial raises several questions. Firstly, follow-up time was only 5.2 years in this trial, and consequently, information on long term effects is lacking. Secondly, despite the fact that women taking HRT at the time of diagnosis had a larger extent of lymph node positive disease and more advanced clinical stage, mortality from CRC was not decreased in this category. Therefore, the clinical relevance of these results needs to be further discussed.

Concerning the effects of combined versus unopposed estrogen therapy, results are diverging. The WHI presented no risk reduction of unopposed estrogen, which is in line with the findings by Newcomb et al. [37], who reported an inverse association between CRC risk and current combined HRT in a large case–control study, but no association with unopposed estrogen therapy. In contrast to these findings, both the California Teacher Study and Campbell et al. reported a lower risk of CRC among ever users of HRT, but no difference in risk by formulation [20].

Few previous studies have addressed the possible interaction between hormone therapy use and CRC risk in relation to clinical disease stage at diagnosis. Grodstein et al. reported a similar risk reduction with hormone therapy for higher and lower stages [43]. In the California Teachers study, the association between HRT and reduced CRC risk was stronger for more advanced stages [6]. However, in both the California Teachers Study, and the Womens Health Trial, the difference in clinical stage at time of diagnosis may be due to the high level of screening-detected cases among these patients, hence being in an earlier stage at diagnosis. In the present study, we found a significantly reduced risk of T stage 1 and 2 tumours among current users of CHRT, and for rectal cancers this risk reduction was also seen for lymph node negative and non-metastatic disease, i.e. less advanced clinical stages.

The relationship between postmenopausal hormone therapy and molecular subtypes of CRC has not yet been thoroughly studied [15,44]. However, a recent study investigated the relationship between HRT use and MSI, BRAF and CIMP status of the tumours [15], whereby HRT (ever vs never use) was inversely associated with overall CRC risk, lower risk for MSI-L/MSS tumours and borderline significantly lower risks for CIMP-negative and BRAF-wildtype tumours, suggesting that HRT may have more pronounced inhibitory effects on the”traditional” pathway, as compared to the serrated or alternate pathways, of colorectal carcinogenesis. Additionally, Lin et al. found no associations between HRT and CRC risk according to MSI or p 53 status [44]. In this present study, we did not find any associations between current use of HRT and risk of CRC according to molecular features of the tumours in the overall analysis. However, as already mentioned, in stratified analysis according to tumour location, we found a lower risk of cyclin D1 and p53 negative tumours in the rectum.

When evaluating the associations between ever use of OC and CRC risk, we found no significant results in the analyses adjusted for established risk factors of CRC. However, in the unadjusted analysis, a significant risk reduction was seen for OC use and all CRC subgroups, except lymph-node positive disease, negative nuclear beta-catenin expression and MSS tumours. The analysis was repeated including one additional covariate in separate models. The association between OC and all studied subgroups remained statistically significant in all models except the one including age at baseline, and also after inclusion of menopausal status in the analysis (data not shown).

For cancers located in the colon, contrasting associations were found, with an increased risk of lymph node-positive and non-metastatic tumours, as well as for cyclin D1 positive tumours.

The epidemiologic evidence for a causal link between OC and CRC risk has not been consistent [19-22,24-26]. To our knowledge, only two previous studies have adressed the question whether this potential association is influenced by molecular features of the tumours. Newcomb et al. have previously studied the association between HRT and MSI status of the tumours and found no association with MSI high tumours, but a risk reduction of 20% for MSI-low/MSS tumours [16,45], and no difference in risk according to tumour location. Additionally, Slattery et al. demonstrated a lower risk of MSI positive tumours among both recent HRT users and OC users [16].

Based on results from emerging translational research, several molecular models have been described to account for the clinicopathological heterogeneity in colorectal carcinogenesis [10-12,34,46,47]. The potential mechanisms by which hormone therapy use reduces risk of CRC development remain unclear. Experimental studies on mice and cell lines have shown that oestrogen- or progesterone-activated signaling leads to growth inhibiting effects on colon cancer cells by upregulating several cell cycle regulators such as p 21, p27 and p53 [48,49]. It has also been proposed that estrogen treatment maintains genomic stability in colonic epithelial cells through upregulation of mismatch repair genes [16,50]. Epigenetic events may also play an important role, as estrogen may be a key factor in the pathway leading to the CpG-island hypermethylation (CIMP) phenotype [51].

General strengths of our study include the population-based, prospective design, the ability to control for multiple potential confounding factors in multivariate risk models and the molecular pathological epidemiology approach linking life style exposures to different tumour markers [14].

Information on HRT use was provided from two different sources, a diary as well as a questionnaire, thus optimising the opportunity to include all HRT-users and minimising the number of under-reporters. We consider our data on HRT to be valid and reliable. This study used two HRT categories, namely current and non-users, which is a limitation as the non-user cohort probably included former users. Such a misclassification is likely to lead to an attenuation of risks and, if anything, observed risks may be underestimated. Regarding exposure to OC, the risk of misclassification is probably lower, since we have used ever vs never use, and most women are peri- or postmenopausal at baseline and will most probably not start treatment with oral contraceptives by that time. Information on duration of exposure of both HRT and OC is lacking in this study, which may have provided deeper knowledge on the associations with CRC risk.

A limitation to the present study is the relatively small number of cases in some subgroups, particularly in the analyses stratified for tumour location. This generates large confidence intervals and, subsequently, low statistical power and the possibility that some of the non-significant findings may have been caused by a potential type II error. Therefore, we regard our study as primarily explorative. To our best knowledge, no studies have described the associations of HRT and OC use and risk of CRC defined by the herein investigated biomarkers. Furthermore, we have no à priori hypothesis on the direction of the possible associations. Given the discovery-driven nature of this study, we consider it important not to increase the risk of a potential type II error by adjusting p-values. Instead, any identified associations will need confirmation in future studies.

Although there are several challenges to molecular epidemiological pathology studies, for example multiple hypothesis testing, misclassification of tumour endpoints, and sample size, the discipline also has unique strengths. MPE can provide insights into mechanisms related to both CRC initiation and progression, in order to optimize personalised prevention and therapy strategies [52]. By interdisciplanary collaboration and education of epidemiologists in molecular pathology, and pathologists in epidemiology and biostatistics, this interdisciplinary field of MPE can be further elaborated. Along with the well described CIN, MSI and DNA methylation pathways of colorectal carcinogenesis, it has further been proposed that other systems and pathways are involved in the pathogenesis of CRC, such as inflammation, and that microRNAs can actively contribute to the carcinogenic process [53].

## Conclusions

In conclusion, our findings provide information on the relationship between use of hormone replacement therapy and oral contraceptives, and risk of colorectal cancer according to several clinicopathological and molecular subsets of the disease, also depending on tumour location. These findings underline the need for continous investigation and further translational research addressing the effects of hormone therapy on key molecular events underlying the different pathways of colorectal carcinogenesis.

## Abbreviations

CRC: Colorectal cancer; HRT: Hormone replacement therapy; CHRT: Combined hormone replacemnet therapy; ERT: Oestrogen replacement therapy; OC: Oral contraceptive; MSI: Microsatellite instability; MSS: Microsatellite stability; CIMP: CpG island methylator phenotype; MDCS: Malmö Diet and Cancer Study; BMI: Body mass index; TMA: Tissue microarray.

## Competing interests

The authors declare that no competing interests exist.

## Authors’ contributions

JB performed all the statistical analyses and drafted the manuscript. SW collected clinical data and evaluated the immunohistochemical stainings. BN constructed the TMAs and carried out all IHC stainings. JE assisted with the collection of clinical data. KJ carried out the histopathological re-evaluation, evaluated the immunohistochemistry, and helped draft the manuscript. JM conceieved of the study, helped with the statistical analyses and helped draft the manuscript. All authors read and approved the final manuscript.

## Pre-publication history

The pre-publication history for this paper can be accessed here:

http://www.biomedcentral.com/1471-2407/14/371/prepub
